# CircCD44 plays oncogenic roles in triple-negative breast cancer by modulating the miR-502–5p/KRAS and IGF2BP2/Myc axes

**DOI:** 10.1186/s12943-021-01444-1

**Published:** 2021-10-25

**Authors:** Jie Li, Xinya Gao, Zhanqiang Zhang, Yuanhui Lai, Xunxun Lin, Bo Lin, Maoguang Ma, Xiaoli Liang, Xixi Li, Weiming Lv, Ying Lin, Nu Zhang

**Affiliations:** 1 Department of Thyroid and Breast Surgery, The First Affiliate Hospital, Sun Yat-sen University, No 58, Zhongshan 2 Road, Guangzhou, 510080 Guangdong Province China; 2 Department of Neurosurgery, The First Affiliate Hospital, Sun Yat-sen University, No 58, Zhongshan 2 Road, Guangzhou, 510080 Guangdong Province China; 3 Department of Plastic Surgery, The First Affiliate Hospital, Sun Yat-sen University, Guangzhou, China

**Keywords:** circRNAs, TNBC, circCD44, KRAS, MYC, IGF2BP2

## Abstract

**Background:**

Emerging studies have revealed the potent functions of circRNAs in breast cancer tumorigenesis. However, the biogenesis, biofunction and mechanism of circRNAs in triple-negative breast cancer (TNBC) are largely unknown.

**Methods:**

High-throughput RNA sequencing was applied to identify dysregulated circRNAs in TNBCs and paired normal tissues. RNA pulldown and luciferase assays were performed to investigate the interaction between circular CD44 (circCD44, also annotated as hsa_circ_0021735) and miR-502–5p. RNA pulldown and RIP assays were used to investigate the interaction between circCD44 and IGF2BP2. Cell viability, colony formation, migration/invasion assays and in vivo tumorigenesis were used to investigate circCD44 biological functions.

**Results:**

CircCD44 is an uncharacterized circRNA, which is highly expressed in TNBC, and its expression is negatively correlated with the prognosis of TNBC patients. CircCD44 promotes TNBC proliferation, migration, invasion and tumorigenesis at least partially by sponging miR-502–5p and interacting with IGF2BP2.

**Conclusion:**

Our data suggested that overexpressed circCD44 promotes TNBC progression. CircCD44 is potentially a novel diagnostic and therapeutic marker for TNBC patients.

**Supplementary Information:**

The online version contains supplementary material available at 10.1186/s12943-021-01444-1.

## Background

Breast cancer is the most common malignancy in women, with a rapidly increasing incidence [1]. Triple-negative breast cancer (TNBC) accounts for approximately 15% of all breast cancers, with the highest recurrence, metastasis, and mortality rates and a lack of effective targeted treatment options [1]. Characterized by the absence of estrogen receptor (ER), progesterone receptor (PR), and human epidermal growth factor receptor 2 (HER2) expression, TNBCs are insensitive to endocrine therapies, molecular targeted therapies and current chemotherapeutics, thus increasing the difficulty of clinical treatment [2, 3]. Although many reagents targeting signaling, such as phosphoinositide 3-kinase (PI3K), cyclin-dependent kinases (CDKs) and receptor tyrosine kinases (RTKs), have been approved by the Food and Drug Administration (FDA), their therapeutic efficacy is still disappointing [4], and there is an urgent need to identify novel molecular markers for early diagnosis or targeted therapies for TNBCs.

Recently, several newly identified biomarkers, such as tumor-associated macrophages (TAMs) [5], microRNAs (miRNAs) [6, 7], and long noncoding RNAs (lncRNAs) [8], have proven to have important prognostic value in TNBCs. Specifically, circular RNAs (circRNAs) have attracted our attention due to their key roles in human cancers, including TNBC. CircRNAs are closed single-stranded circular transcripts with no 5′ caps or 3′ poly(A) tails [9]. CircRNAs have been reported to be distributed widely across species and to play crucial roles, such as miRNA sponges, RNA binding proteins (RBPs) and protein coding templates, in tumorigenesis and cancer progression. Given their unique biological structure and vital functions, looking for circRNAs with high specificity and sensitivity will provide a new opportunity for the early diagnosis, clinical treatment, and prognosis monitoring of TNBC [10, 11]. To date, several TNBC-related circRNAs have been reported. CircSEPT9, induced by E2F1 and EIF4A3, promotes TNBC tumorigenesis and serves as a progression marker [12]. Another circRNA, circHER2, encodes a polypeptide and resensitizes TNBC to pertuzumab-based treatment [13]. Nevertheless, the roles of circRNAs in TNBC remain largely illusive.

In this study, we aimed to identify new TNBC biomarkers that predict the prognosis for clinical patient management and novel targets for precise therapy. We determined that circCD44 promotes TNBC progression via miR-502–5p/KRAS and IGF2BP2/C-Myc signaling and suggested that circCD44 is a potential therapeutic target for TNBCs.

## Methods

### Human samples

All samples were obtained from the Department of Breast and Thyroid Surgery of the First Affiliated Hospital of Sun Yat-sen University. Written and informed consent was obtained. The research was approved by the Ethics Committee of the First Affiliated Hospital of Sun Yat-sen University.

### RNA sequencing

RNA sequencing was applied as previously described [13]. Briefly, total RNA was extracted, purified, and reverse transcribed. CircRNA junction data were downloaded from circBase (http://www.circbase.org/). CircRNAs with a fold change > 2, *p* < 0.01, FDR < 0.05 were identified as significantly differentially expressed circRNAs. The RNA seq data access number is NCBI SRP266211, totally, 1200 differentially expressed circRNAs were identified and the consistent up- or down- regulated circRNAs in 5 TNBCs were listed in Supplementary Table 4.

### Cell lines

The human normal breast epithelium MCF-10A and human mammary cancer cell lines Hs578T, BT-549, MDA-MB-468 and MDA-MB-231 were obtained from the American Type Culture Collection (ATCC, Manassas, VA). Cells were cultured in Dulbecco’s modified Eagle’s medium (DMEM), minimum essential medium (MEM) and RPMI-1640 and supplied with 10% fetal bovine serum (Life Technologies, Grand Island, NY, USA), 1% penicillin G, and streptomycin.

### Plasmid and stable cell line construction

The circCD44 plasmid was generated by chemical synthesis of the complete sequence of circCD44, and additional circularization promoter ALU sequences were added upstream and downstream [14],the sequence was detailed as below: 5’TCTGACAACTGAACTGCTCTCGCCTTGAACCTGTTTTGGCACTAA AATAAAATCTGTTCAATTAACGAATTCTGAAATATGCTATCTTACAG----GTGAATATATTTTTTCTTGAGGATCCACTAATTTGGGATGATAACGCCAAAACAGGTTCAAGGCGAGAGCAGTTCAGTTGTCAGAA3’,the sequence of circCD44 was cloned between AG and GT in the blank. Mutant circCD44 was established by changing the interaction residue CAAGAA to GUUCUU, as shown in Fig. 4A. In Fig. 6F, circCD44-mut was generated by changing the interacting residues mentioned in Fig. 6E: U to A, A to U, C to G and G to C. The IGF2BP2 mutant allele was established by changing the interaction residues to IMKDNKHSDCDRQDT. The circCD44 shRNA-1 sequence was GAAGGATGGTCCAGGCAACTC, and the shRNA-2 sequence was AGAAGGATGGTCCAGGCAACT. The plasmids were transfected with Lipofectamine 3000 (Invitrogen, Carlsbad, CA, USA) according to the manufacturer’s instructions.

### Antibodies

The antibodies used in this paper are detailed below:

N-cadherin (CST, cat# 13116), E-cadherin (CST, cat# 14472), PCNA (CST, cat# 13110), Slug (CST, cat# 9585), Snail (CST, cat# 3879), vimentin (CST, cat# 5741), β-actin (CST, cat# 3700), KRAS (CST, cat# 67648), p-AKT (473) (CST, cat# 4060), p-AKT (CST, cat# 9275), IGF2BP2 (CST, cat# 14672), HA (CST, cat# 5017), CD44 (BD Pharmingen; IM7) and C-myc (CST, cat# 18583).

### Cell proliferation assay

A Cell Counting Kit-8 (CCK-8, Dojindo, Tabaru, Japan) was used to measure cell proliferation. Cells were seeded into 96-well plates, collected at different time points, and absorbance at 450 nm was detected. The relative level was then obtained by normalization from at least three independent experiments.

### Colony formation assay

To investigate anchorage-independent effects on proliferation, a specified number of cells were seeded in a six-well plate. After incubation for 14 days, the colonies were stained with crystal violet (Sigma–Aldrich, St. Louis, MO, USA), and the colonies were counted.

### Apoptosis assay

Cells with the indicated modifications were subjected to apoptosis assays following the assay kit (Invitrogen™ V35113) manufacturer’s instructions. This flow cytometry product detects the externalization of phosphatidylserine in apoptotic cells using recombinant annexin V conjugated to red laser-excited allophycocyanin, and dead cells using Green nucleic acid stain. Briefly, Apoptotic cells are detected by annexin V binding to externalized phosphatidylserine, and late apoptotic and necrotic cells have compromised membranes that permit green stain access to cellular nucleic acids. Cells with indicated modifications were subjected to apoptosis assay and live cells show little or no fluorescence, apoptotic cells show red fluorescence and very little green fluorescence, while late apoptotic cells show a higher level of red and orange fluorescence. Cells were distinguished using a flow cytometer that has both 488 nm and 633 nm excitation sources.

### EdU assay

For the EdU assay, cells were seeded into 6-well plates and cocultured with an EdU-labeling reagent (Beyotime, C0071S) after incubation for 2 h. The cells were fixed and stained following the manufacturer’s protocol. Five fields of view were evaluated for each cell line.

### Dual luciferase activity reporter system

The Renilla luciferase (Rluc) and firefly luciferase (Luc) sequences were amplified from the psiCheck 2 vector (Promega, USA). Rluc was placed in the upstream position, and Luc was placed in the downstream position. The KRAS sequence along with its 3′ UTR was amplified and inserted between Rluc and Luc. Relative activity was determined by normalization to Rluc and control.

### LC–MS analysis

Proteins were separated via 12% SDS–PAGE and subjected to digestion with sequencing-grade trypsin (Promega, Madison, WI, USA). The fragments were analyzed using the National Center for Biotechnology Information nonredundant protein database with Mascot (Matrix Science, Boston, MA, USA) to identify specific peptides. The top 50 potential interacting proteins identified by LC/MS are shown in Supplementary Table 3.

### Flow cytometry

Whole blood samples were collected from mice 5 weeks after establishing pulmonary metastatic models. Peripheral blood mononuclear cells (PBMCs) were enriched from the blood as a control. GFP-tagged tumor cells were selected using the selected GFP gate. Flow cytometric analysis was performed on a flow cytometer FACSCalibur (BD Biosciences, San Jose, CA).

### Animal studies

Four-week-old female BALB/c nude mice were purchased from the Laboratory Animal Center of Sun Yat-sen University. All animals were treated in accordance with the guidelines of the Committee on Animals of Sun Yat-sen University. A total of 5 × 10^6^ cells were inoculated into the mammary fat pad to establish subcutaneous xenografts. A total of 5× 10^3^ cells were injected through the caudal vein. After 6 weeks, the samples were harvested and subjected to HE staining.

### Immunohistochemistry (IHC)

Tumor slices were cut to an 8 to 10-μM thickness. After being deparaffinized in xylene and rehydrated, the tissue sections were washed 3 times for 10 min with PBS and incubated for 1 h in goat serum dilution buffer at room temperature. Primary antibodies were applied overnight at 4 °C in a wet chamber. After washing three times for 10 min with wash buffer, the tumor sections were incubated with secondary antibodies for 60 min at room temperature. The tumor sections were subsequently washed three times with the above wash buffer. Diaminobenzidine (DAB) reagent was added to these tumor sections, which were then counterstained with hematoxylin to visualize the nuclei.

### RNA fluorescence in situ hybridization (FISH)

Cells were incubated with 50% formamide, 2X SSC, 0.25 mg/mL *Escherichia coli* transfer RNA, 0.25 mg/mL salmon sperm DNA (Life Technologies), 2.5 mg/mL BSA, and fluorescently labeled junction probe for 12 h. The cells were then washed and incubated overnight at room temperature. Images were captured using confocal microscopy. The sequence of the detection probe was as follows: 5′ cy3-TAGGAGTTGCCTGGACCATCCTTCTTCCTG 3′.

#### Reverse transcription and real-time (RT) PCR

Total RNA was extracted with a PureLink RNA mini kit (Thermo Fisher Scientific). After reverse transcription, cDNAs were harvested, and the resulting cDNA was then subjected to real-time PCR analysis with SYBR Select Master Mix (Thermo Fisher Scientific) in a StepOne Plus real-time PCR system (Applied Biosystems). The results for each sample were normalized to β-actin mRNA. The key primers are listed in Supplementary Table 2.

#### Northern blotting

Twenty micrograms of total RNA was extracted and separated using 1.2% agarose gel electrophoresis. After nelon membrane permeabilization and fixation, specific probes were applied at 42 °C and washed with 0.1% SDS at 68 °C. The circCD44 detection probe was as follows: 5′ CTACTAGGAGTTGCCTGGACCATCCTTCTTCCTGCTTGA –DIG 3′.

### RNA pulldown

The cells were harvested, lysed and incubated with beads at 4 °C overnight. The beads were coated with biotin tagged circRNA using Pierce™ RNA 3′ End Desthiobiotinylation Kit (Thermo 20,163). After washing with wash buffer, RNA-Protein complex was harvested, the RNA complex was purified with TRIzol reagent and then subjected to qRT–PCR analysis. The complex was incubated with loading buffer to separate the protein from the complex and subjected to immunoblot assay and LC/MS assay.

### RNA immunoprecipitation

RNA immunoprecipitation (RIP) was performed using a Magna RIP Kit (Millipore) following the manufacturer’s protocol. Cells were lysed with lysis buffer and incubated with conjugated beads for 6 h at 4 °C. After treatment with proteinase K, the protein was removed. RNA was extracted and purified with TRIzol Reagent. The purified RNA was subjected to qRT–PCR for further analysis.

### Western blotting

Equal amounts of protein were added to each well containing 12% SDS–PAGE gels. After separation and transfer to membranes, the bands were blocked with 5% FBS and incubated with the corresponding primary antibody at 4 °C overnight. After incubation with the secondary antibody, the bands were visualized with an ECL kit.

### Molecular docking

The X-ray structure of IGF2BP2 was downloaded from the RCSB Protein Data Bank (PDB code: 6ROL). HDOCK3 was used for docking IGF2BP2 and RNA; RNA was selected as the receptor, and IGF2BP2 was selected as the ligand. The interaction of the ligand and receptor was analyzed in Molecular Operating Environment (MOE) v2014.094 and visualized with PyMOL (www.pymol.org).

### RNA stability assay

The cells were seeded into 6-well plates and grown to 50% confluence. They were then treated with 5 μg/ml actinomycin D and collected at the indicated points. RNA levels were detected using qRT–PCR, and the half-life of mRNA was evaluated according to a published paper [15].

### Statistical analysis

Statistical analysis was applied using GraphPad Prism software. The data are presented as the mean ± SD. As indicated, Student’s two tailed unpaired t test was used to determine the statistical significance of the in vitro experiments. The log-rank test or Gehan-Breslow-Wilcoxon test was used to determine significant differences in the survival data. A *p* value of less than 0.05 was considered statistically significant. For each experiment, data are representative of at least three replications with similar results.

## Results

### CircCD44 was highly expressed in TNBCs, and its expression was negatively correlated with patient prognosis

**Fig. 1:**
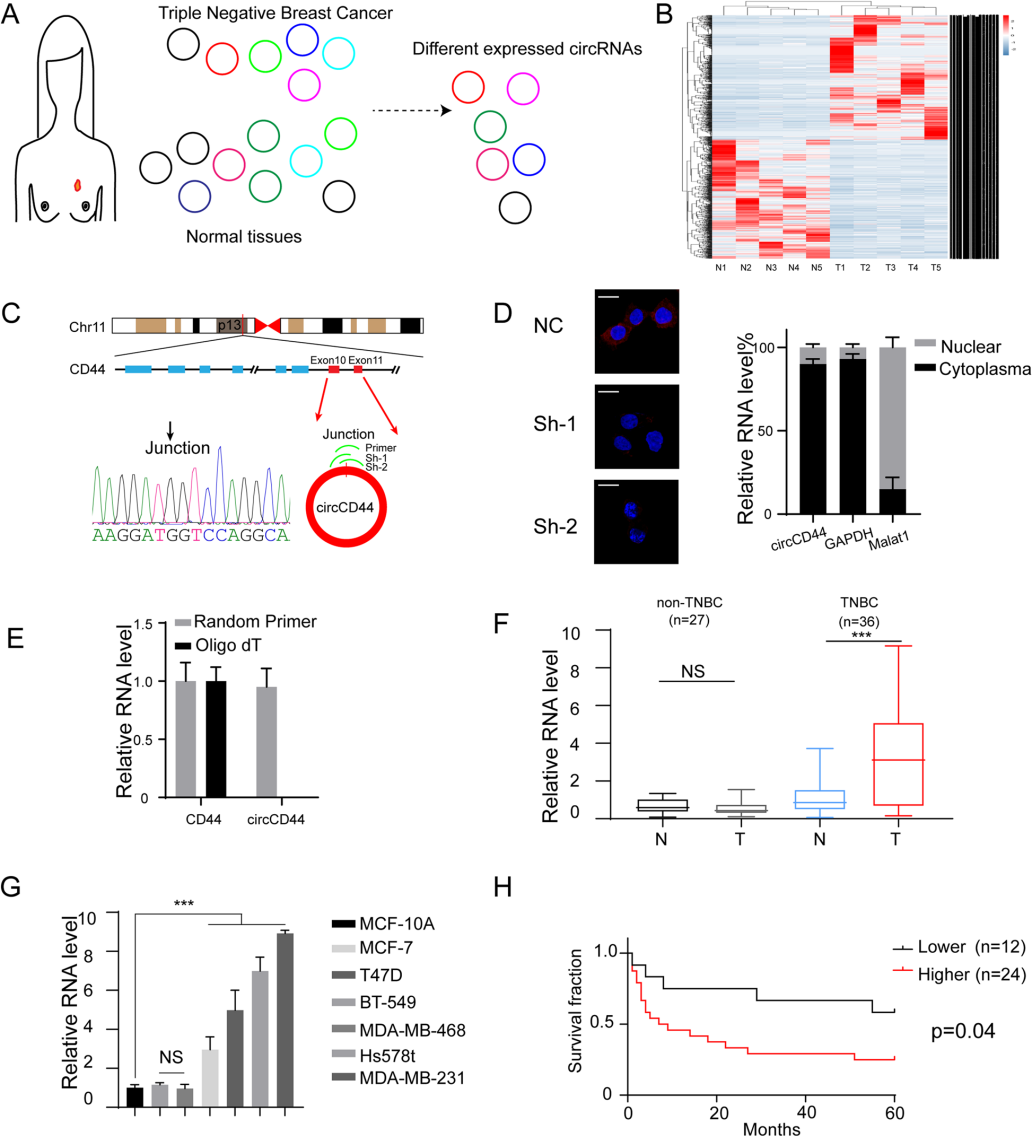
CircCD44 was upregulated in triple-negative breast cancers (TNBCs). **A** Schematic graph of the scanning strategy. Five paired normal tissues and TNBCs were included and subjected to circRNA sequencing to identify the dysregulated circRNAs, as we previously reported [13]. **B** Heatmap of candidate RNA sequences in TNBCs and paired normal tissues. Blue, downregulated circRNAs; red, upregulated circRNAs. **C** Schematic diagram of circCD44 formation from CircBase; validation strategy for circCD44 and Sanger sequencing of the junction of circCD44. **D** Left: FISH detection of circCD44 using a junction-specific probe in the indicated shRNA-transfected MDA-MB-231 cells; scale bar: 20 μm. Right: qRT–PCR analysis of circCD44 in different cell fractions. GAPDH and Malat1 were used as positive controls for the cytoplasm and nuclei. **E** qRT–PCR analysis of CD44 and circCD44 using random primers or oligo dT primers. **F** Relative RNA level of circCD44 in 36 TNBCs and 27 non-TNBCs and paired normal tissues. All RNA levels were normalized to the paired normal tissue of TNBC; ***, *P* < 0.001, NS, non-significant. **G** Relative circCD44 level in breast cancer cell lines and normal breast epithelial cells; ***, *P* < 0.001. **H** Thirty-six TNBC patients were divided into two groups with a cutoff of the mean level of circCD44 detected by qRT–PCR in the whole cohort. Kaplan–Meier survival analysis was then applied. Data are representative of at least 2–3 experiments with similar results

Five pairs of TNBCs and adjacent normal tissues were collected and subjected to circRNA sequencing as we previously described (dataset access number: NCBI SRP266211) [13] (Fig. 1A). Among the differentially expressed circRNAs, circCD44 (hsa_circ_0021735) was the most upregulated circRNA in TNBCs compared with normal tissues (Fig. 1B). CircBase revealed that circCD44 was generated from exon 10 to exon 11 of *CD44*. Sanger sequencing confirmed the junction sequence in the divergent primers spanning the predicted products (Fig. 1C). To investigate the cellular location of circCD44, we generated two specific junction shRNAs, transfected them into the MDA-MB-231 cell line and subjected these cells to FISH detection with a junction-specific probe. We observed that circCD44 was mostly located in the cytoplasm and that shRNA markedly knocked down the expression of endogenous circCD44, which was also confirmed in TNBC clinical species (Fig. 1D, left; Supplementary Figure 1A). Furthermore, the cell fraction qRT–PCR assay confirmed this observation (Fig. 1D, right). Northern blotting using a junction-specific probe indicated that circCD44 was resistant to RNase R^+^ digestion and validated the specificity of the shRNAs and circCD44 overexpression plasmid (Supplementary Figure 1B).

We found that only random primers spanned circular products, while Oligo dT could not amplify circCD44 (Fig. 1E). We next detected the circCD44 expression pattern in patient cohorts including 36 TNBCs and 27 non-TNBCs and their adjacent normal tissues. The results showed that circCD44 was overexpressed in TNBCs but not in non-TNBCs compared with normal tissues, indicating that circCD44 may be a TNBC subtype-specific circRNA (Fig. 1F, NS, not significant, ***, *p* < 0.001). We also determined the relative expression level of circCD44 in non-TNBC and TNBC cell lines, and the results showed that circCD44 was also overexpressed in TNBC cell lines but not in non-TNBCs (MCF-7, T47D) (Fig. 1G, NS, not significant, ***, *p* < 0.001). When the 36 TNBCs were divided into two groups with the mean circCD44 expression of the whole cohort of TNBCs as the cutoff, we found that circCD44 expression was significantly negatively correlated with the prognosis of TNBC patients (Fig. 1H, *p* < 0.001). These results suggested that circCD44 is specifically expressed in TNBC and predicts worse patient prognosis.

### CircCD44 promotes the malignancy characteristics of TNBCs

**Fig. 2:**
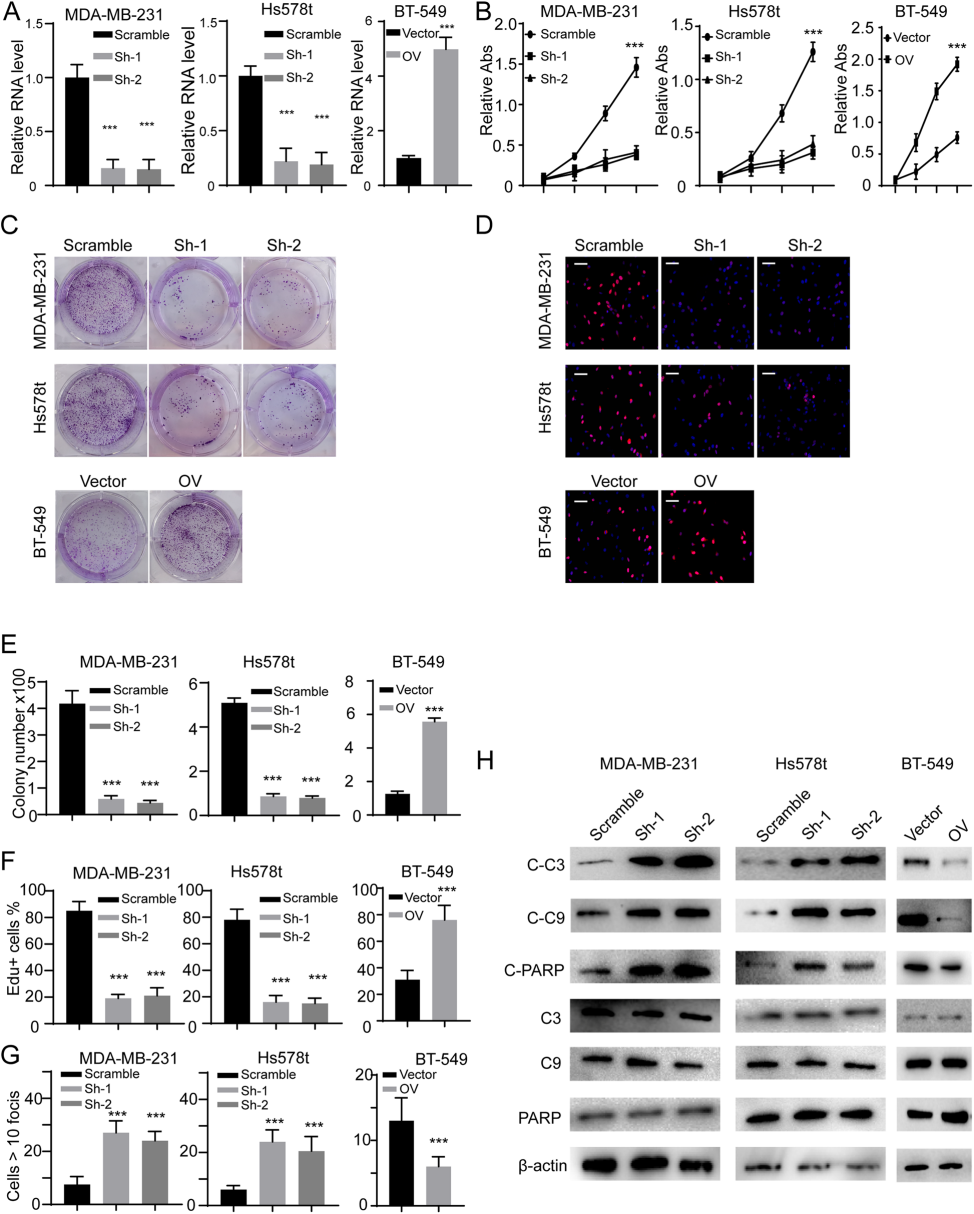
CircCD44 promotes the proliferation and chemoresistance of TNBCs. **A** Establishment of circCD44-stable knockdown and overexpression cell lines using two specific shRNAs or circCD44 plasmid. The relative RNA level was detected in each cell line; ***, *P* < 0.001. **B** CCK-8 assay of different cell lines with the indicated modifications; ***, *P* < 0.001. **C** Representative images of colony formation assays in different cell lines with the indicated modifications. Statistical analysis is shown in (**E**); ***, *P* < 0.001. **D** Representative image of the EdU assay in different cell lines with the indicated modifications. Statistical analysis is shown in **(F)**. Scale bar: 50 μm; ***, *P* < 0.001. **G** The indicated cells were treated with Cyc, and the rate of apoptosis was detected; ***, *P* < 0.001. **H.** Immunoblot of the caspase cascade in different cell lines with the indicated modifications. C-C3, cleaved Caspase 3; C-C9, cleaved Caspase 9; C3, Caspase 3; C9, Caspase 9. Data are representative of at least 2–3 experiments with similar results

**Fig. 3:**
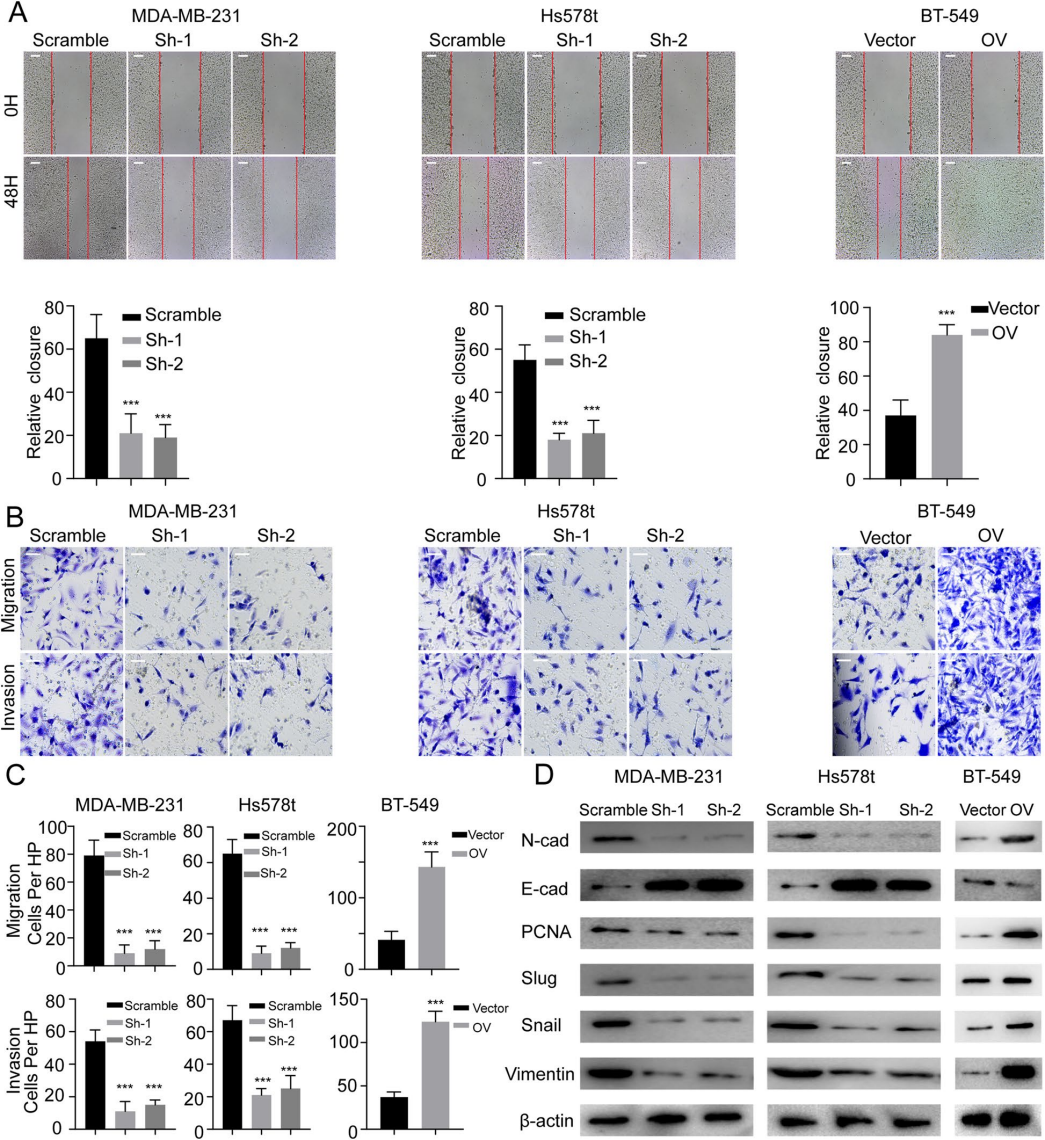
CircCD44 promotes the migration and invasion of TNBCs. **A** Representative image of the wound healing assay (upper) and relative wound closure (lower) in TNBCs with the indicated modifications, scale bar: 100 μm; ***, *p* < 0.001. **B** Representative image of the migration assay and invasion chamber assay in TNBCs with the indicated modifications; scale bar: 50 μm. **C** Statistical analysis of migration assay and invasion chamber assay in TNBCs with the indicated modifications; ***, *P* < 0.001. **D** Immunoblot of EMT markers in indicated cells. Data are representative of at least 2–3 experiments with similar results

To study the unknown functions of circCD44, we generated both stable circCD44 knockdown and circCD44 overexpression cell lines (Fig. 2A, ***, *p* < 0.001). We found that knocking down circCD44 inhibited MDA-MB-231 and Hs576t cell proliferation, while ectopic expression of circCD44 promoted BT-549 cell growth (Fig. 2B-F, ***, *p* < 0.001). We also performed apoptosis assays and observed that depletion of circCD44 could promote breast cancer cell apoptosis, as detected with cleaved PARP or Caspase3 (Fig. 2G, H**,** ***, *p* < 0.001). Importantly, we also observed that circCD44 could improve TNBC cell migration, invasion and chemoresistance (Fig. 3A-C, ***, *p* < 0.001). Since epithelial-mesenchymal transition (EMT) is one of the major causes of cell invasion and migration, we next detected EMT markers and found that mesenchymal markers such as N-cadherin, Slug, and Snail were all decreased, whereas epithelial markers such as E-cadherin were increased upon depletion of circCD44 (Fig. 3D). The above results suggested that circCD44 is an oncogenic circRNA in TNBC and promotes the malignant behavior of TNBC cells.

### CircCD44 directly binds to miR-502–5p and acts as a competitive endogenous RNA

**Fig. 4:**
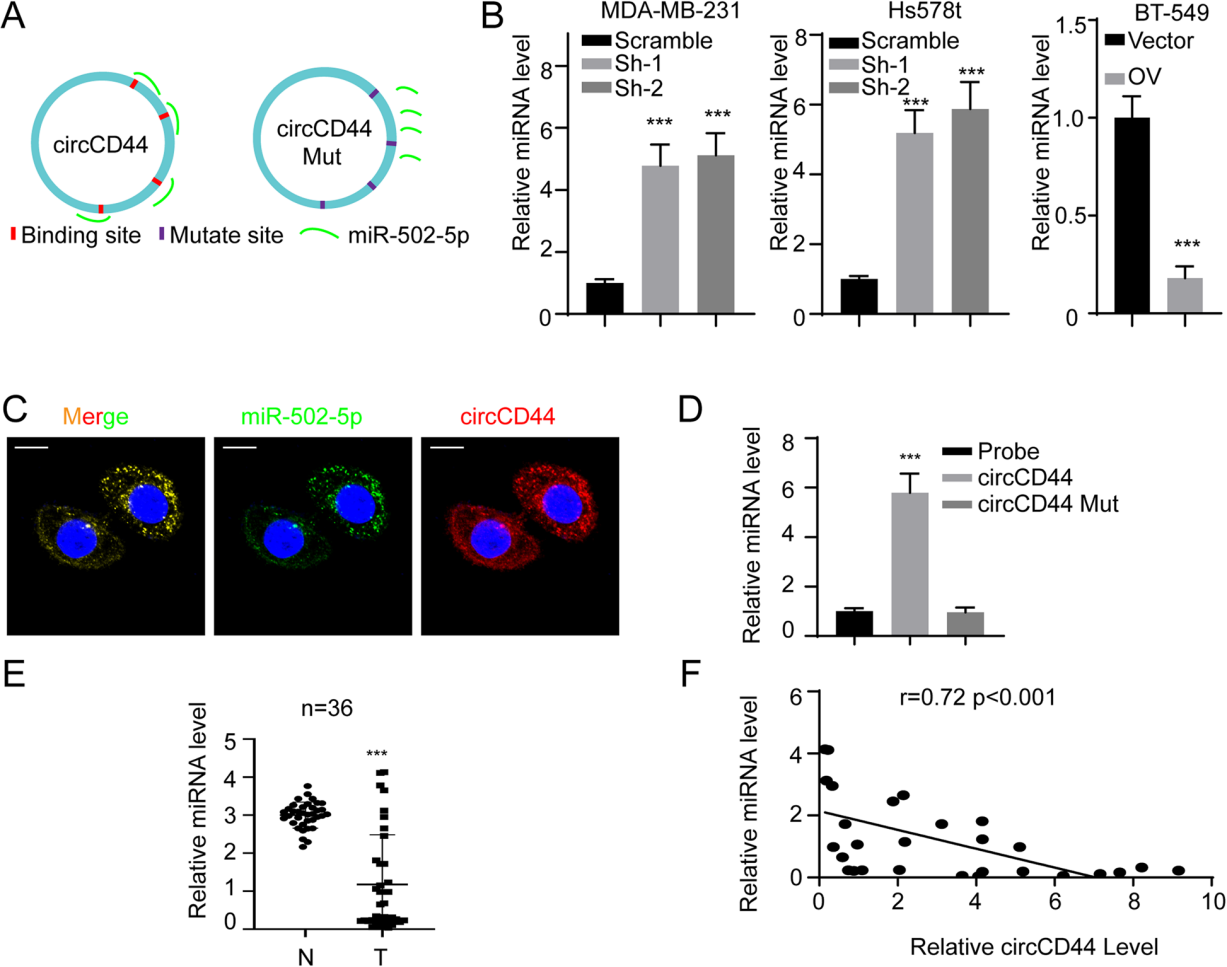
CircCD44 acts as an endogenous competing RNA by sponging miRNA miR-502–5p. **A** Schematic diagram of circCD44, miR-502–5p and the mutant allele. We identified miR-502–5p as a potential target miRNA by searching CircNET, RNAhybrid and miRNAda tools. The interaction site between circCD44 and miR-502–5p was obtained with an online tool (https://circinteractome.nia.nih.gov/). The circCD44 mut allele was established by replacing A with U, U with A, C with G and G with C. **B** Relative RNA level of miR-502–5p in TNBC cell lines with the indicated modifications using miRNA detection primers; ***, *P* < 0.001. **C** FISH of circCD44 and miR-502–5p in the MDA-MB-231 cell line, scale bar: 20 μm. **D** BT-549 cells were transfected with circCD44 and the mutant allele. RNA pulldown was applied using a junction-specific probe, the complex was subjected to qRT–PCR assay, and then miR-502–5p was detected and normalized; ***, *P* < 0.001. **E** The relative RNA level of miR-502–5p was determined in the whole cohort of our in-house TNBC database; ***, *P* < 0.001. **F** The correlation between miR-502–5p and circCD44 was evaluated, and regression analysis was applied (*r* = 0.72, *P* < 0.001). Data are representative of at least 2–3 experiments with similar results

Overexpressed circRNAs in cancer may be due to their amplified host genes [16]. We then detected the expression pattern of CD44 in the TCGA database and the whole in-house cohort. There was no significant difference between normal tissue and tumors in the TCGA database or our in-house cohort (Supplementary Figure 2A, B). We also detected the expression level of CD44 in the cell lines described above. The results showed that CD44 expression remained unaffected in different cell lines, indicating that circCD44 exerts its function independent of CD44 (Supplementary Figure 2C, D). Given its preferential localization in the cytoplasm and lack of potential open reading frame (ORF), we hypothesized that circCD44 could serve as a miRNA sponge to compete with endogenous RNA [16]. To this end, several potential target miRNAs, including miR-502–5p, were identified by employing the CircNET, RNAhybrid and miRNAda tools. Based on the complementary sequences, the mutant circCD44 was established by changing the interaction residue CAAGAA to GUUCUU (Fig. 4A). Consistent with the above finding, knockdown of circCD44 increased, whereas ectopic expression of circCD44 decreased miR-502–5p RNA levels (Fig. 4B, ***, *p* < 0.001). In further FISH assays, colocalization of circCD44 and miR-502–5p in the cytoplasm was observed (Fig. 4C). RNA pulldown assays were also performed, and the results showed that wild-type circCD44 - but not the miR-502–5p-binding deficient mutant - could bind with miR-502–5p, thus indicating that miR-502–5p directly binds to circCD44 at these four potential sites (Fig. 4D, ***, *p* < 0.001). In further investigations of the expression of miR-502–5p in TNBC patients, we observed that miR-502–5p was downregulated in tumors compared with normal tissues (Fig. 4E, ***, *p* < 0.001). More importantly, the expression of miR-502–5p was negatively correlated with circCD44 expression in these TNBC patients (Fig. 4F, r = 0.72, *p* < 0.001).

### CircCD44 inhibits miR-502–5p-mediated KRAS degradation

**Fig. 5:**
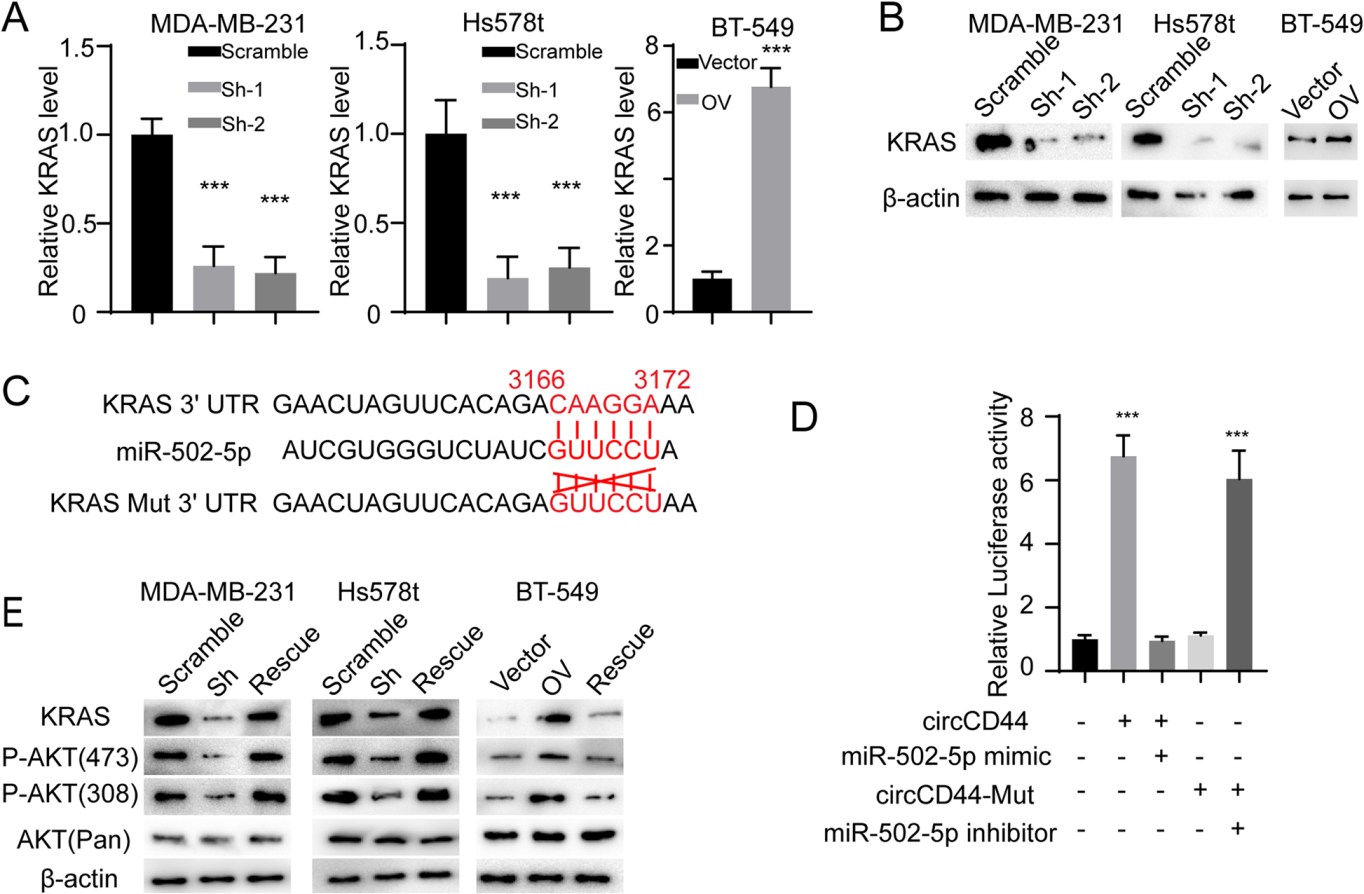
CircCD44 inhibits miR-502–5p-mediated KRAS degradation. **A** The relative KRAS RNA level was detected in different cells with the indicated modifications; ***, *P* < 0.001. **B** Immunoblot of KRAS in cells with the indicated modifications. **C** Schematic image of the binding site between miR-502–5p and circCD44 and the mutant allele. The binding site was obtained from the online tool, TargetScan, and the mutant allele was established as illustrated. **D** HEK293T cells were transfected with different plasmids or microRNAs as indicated. Relative luciferase activity was detected and normalized to the control; ***, *p* < 0.001. **E** miR-502–5p inhibitor was transfected into circCD44 knockdown cells, and miR-502–5p mimic was transfected into circCD44-overexpressing cells (these two cell lines were named “Rescue”). Immunoblot for KRAS and downstream AKT signaling components in TNBCs with the indicated modifications was detected. Data are representative of at least 2–3 experiments with similar results

miRNAs generally exert their biological function by binding to the 3′ untranslated region and CDS of specific premRNAs, leading to premRNA degradation [17]. To determine the downstream effector of circCD44-miR-502–5p, we scanned the target genes of miR-502–5p using the online tool TargetScan [18] and found that KRAS was one of the most vital genes involved in the tumorigenesis and progression of many cancers. To validate our findings, we found that knockdown of circCD44 could markedly decrease KRAS expression, whereas ectopic expression of circCD44 could increase KRAS expression at both the RNA and protein levels (Fig. 5A, B, ***, *p* < 0.001). Furthermore, we established the KRAS Mut allele accordingly (Fig. 5C) to further verify the potential role of circCD44 in KRAS expression. We transfected circCD44, circCD44-Mut, miR-502–5p mimic and miR-502–5p inhibitor into HEK293T cell lines individually or in combination. We observed that circCD44 expression - but not circCD44 and miR-502–5p mimic coexpression or circCD44-mut expression - could enhance KRAS reporter activity (Fig. 5D, ***, *p* < 0.001). We also transfected a miR-502–5p inhibitor into circCD44 knockdown cell lines and a miR-502–5p mimic into circCD44 overexpression cell lines (these two cell lines were named “Rescue” in Fig. 5E) and detected the expression of KRAS and downstream p-AKT. The results showed that depletion of circCD44 could decrease KRAS expression and downstream phosphorylation of AKT, which could be markedly rescued by expression of a miR-502–5p inhibitor. On the other hand, circCD44-induced KRAS expression could be attenuated by expressing a miR-502–5p mimic (Fig. 5E). Together, these findings indicate that circCD44 regulates the KRAS pathway by attenuating miR-502–5p-mediated KRAS degradation.

To measure whether hormonal signaling correlates with the circCD44/miR-502–5p axis, we treated MCF-7 and T47D ER/PR-positive breast cancer cells with estrone and Norgestimate (10 mol/L each) and detected the expression of circCD44 and miR-502–5p. The results are shown in Supplementary Figures 3A and B (NS, non-significance). p-ER and p-PR were also detected for confirmation (Supplementary Figure 3C). The levels of circCD44 and miR502–5p were rarely changed after estrone and Norgestimate treatment. We thus thought that this interacting axis existed only in TNBC cells.

### CircCD44 directly binds to IGF2BP2 in TNBCs

**Fig. 6:**
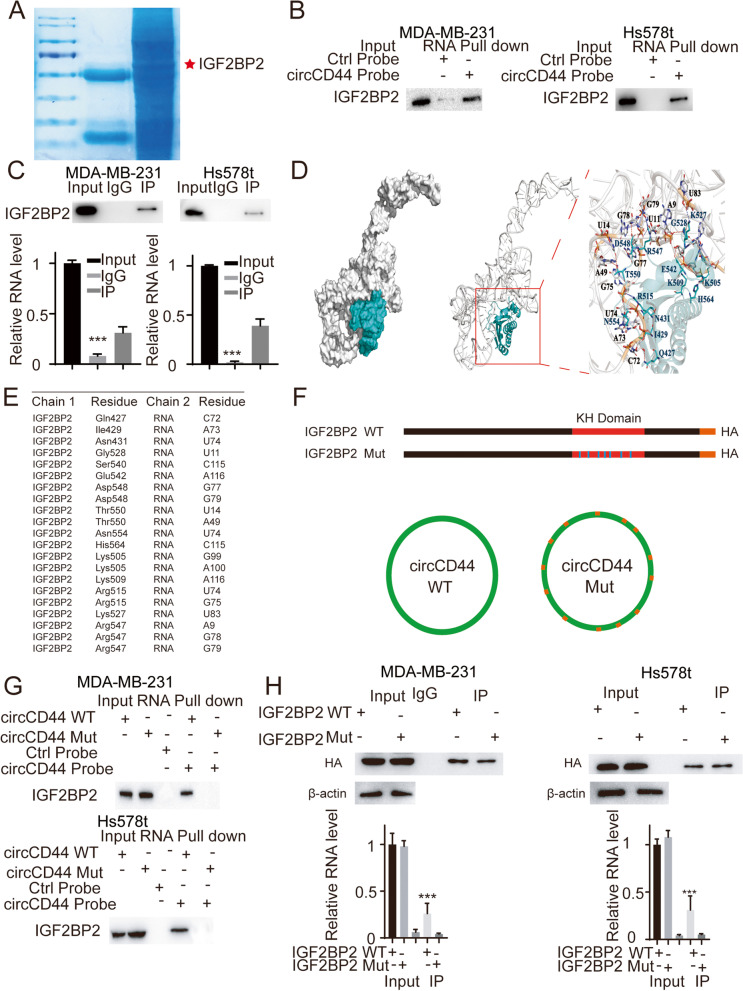
CircCD44 directly binds to IGF2BP2. **A** RNA pulldown and LC–MS assay. An RNA pulldown assay was applied in the MDA-MB-231 cell line using junction-specific probes, and the samples were subjected to LC–MS. IGF2BP2 was identified and indicated. **B** The indicated cells were subjected to an RNA pulldown assay, and IGF2BP2 was detected. **C** An RIP assay was applied using IGF2BP2 antibody in TNBCs, and the complex was subjected to qRT-PCT assay. The relative RNA level of circCD44 was detected, ***, *p* < 0.001. **D** Molecular docking predicting the potential binding motif of circCD44 and IGF2BP2; gray, circCD44; blue, IGF2BP2. **E** Detailed amino acid interacting residues of IGF2BP2 and circCD44 predicted in **(D)**. **F** Schematic diagram of WT and the mutant allele. circCD44 Mut was generated by changing interacting residues U to A, A to U, C to G and G to C. An IGF2BP2 mutant was established by replacing the interacting residue with IMKDNKHSDCDRQDT. **G** MDA-MB-231 cells were transfected with WT/Mut circCD44 and subjected to an RNA pulldown assay. IGF2BP2 was detected with immunoblotting. **H** Cells transfected with WT/Mut IGF2BP2 were subjected to an RIP assay, and the relative RNA level of circCD44 was measured; ***, *p* < 0.001. Data are representative of at least 2–3 experiments with similar results

We established the rescue cell lines described in Fig. 5E and found that although KRAS was completely restored, the malignancy characteristics of TNBCs were only partially restored. Since circRNAs could also perform their functions by directly binding to specific proteins [19], we applied RNA pulldown coupled with LC/MS analysis to characterize potential circCD44-binding proteins (Fig. 6A). Among them, IGF2BP2 was identified and validated by RNA pulldown and RIP assays (Fig. 6B, C, ***, *p* < 0.001). Furthermore, IGF2BP2 molecular docking was performed, and the interaction motif in IGF2BP2 and the binding sites in circCD44 were characterized (Fig. 6D, E). To further investigate their interaction, the IGF2BP2 Mut and circCD44 mut alleles were established accordingly: circCD44-mut was generated by changing the interacting residues mentioned in Fig. 6E (U to A, A to U, C to G and G to C), and an IGF2BP2 mutant was established by changing the interaction residues to IMKDNKHSDCDRQDT (Fig. 6F). The results showed that mutations in both the IGF2BP2 interaction motif and the circCD44 binding sites disturbed the interaction of circCD44 and IGF2BP2 (Fig. 6G, H, ***, *p* < 0.001), thus indicating that circCD44 could directly bind to IGF2BP2.

### CircCD44 promotes the stability of Myc mRNA by affecting IGF2BP2

**Fig. 7:**
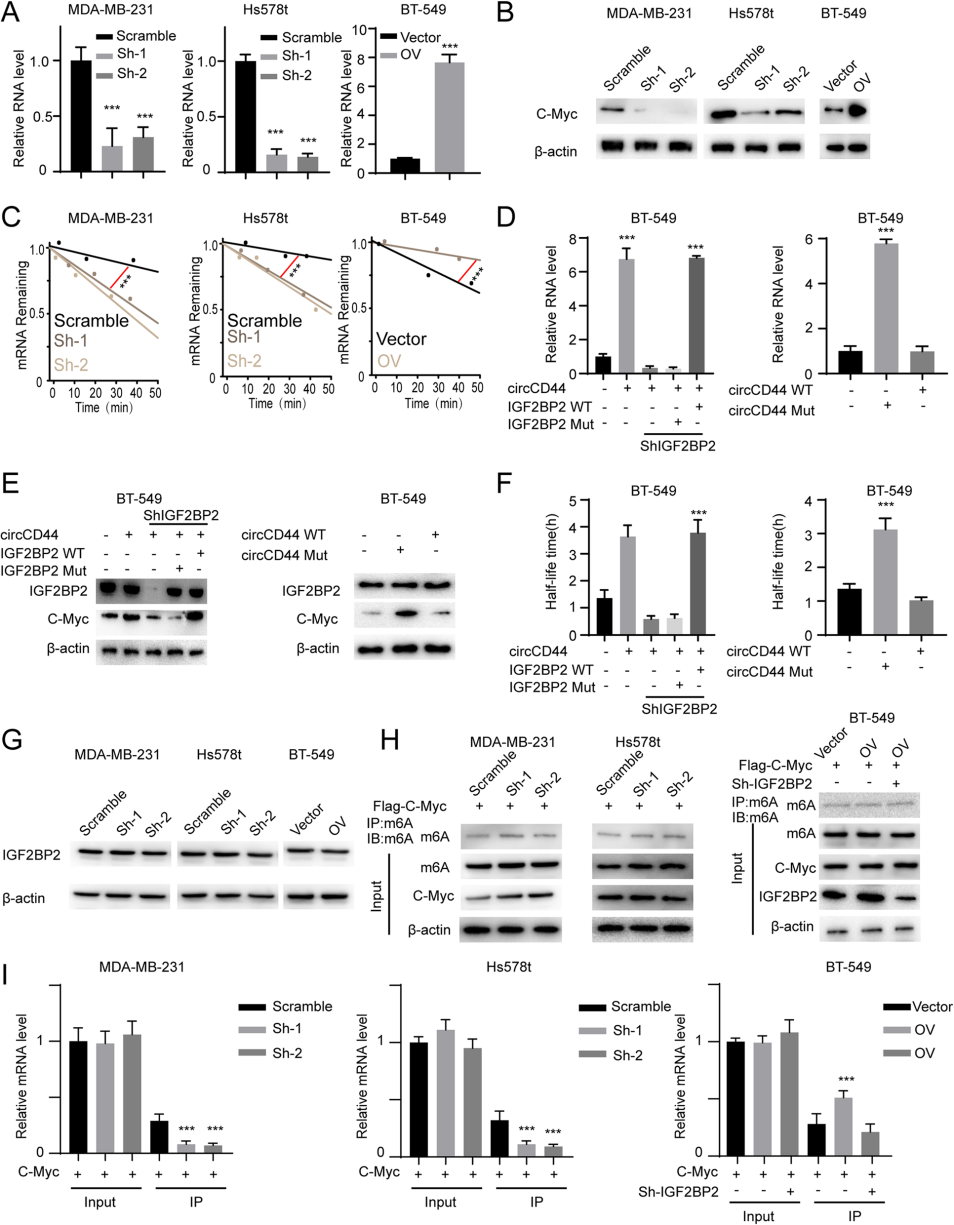
CircCD44 prolonged C-Myc mRNA half-life in an IGF2BP2-dependent manner. **A** Relative C-Myc RNA level in cells with the indicated modification; ***, *p* < 0.001. **B** Immunoblot for C-Myc in cells with the indicated modifications. **C** mRNA half-life of C-Myc in TNBCs with different modifications; RNA was collected at different times and subjected to qRT–PCR assay, and the relative C-myc RNA was detected and normalized, ***, *P* < 0.001. **D** Left: BT549 cells were transfected with IGF2BP2-specific shRNAs, and IGF2BP2 WT/Mut was then transfected to re-express different IGF2BP2 alleles. Right: BT549 cells were transfected with circCD44 WT/Mut. The relative C-Myc RNA level was detected; ***, *p* < 0.001. **E** Immunoblot for IGF2BP2 and C-Myc in BT549 cells with the indicated modifications. **F** Half-life of C-Myc mRNA in BT549 cells with the indicated modifications; ***, *P* < 0.001. **G** Immunoblot for IGF2BP2 in cells with circCD44 knockdown or overexpression. **H** RIP-IB assay. m6A antibody was used for RIP assay, and m6A, C-Myc and IGF2BP2 were measured in cells with the indicated modifications. C-Myc was transfected into all cells to avoid endogenous expression variation. **I** The RIP assay in **(H)** was applied, and the complex was subjected to qRT–PCR assay. The relative RNA level of C-Myc was detected, ***, *p* < 0.001. Data are representative of at least 2–3 experiments with similar results

IGF2BP2 has been reported to stabilize mRNA and thus contribute to the progression of cancers, and Myc was reported to be the key target of IGF2BP2 [15]. Due to the ability of circCD44 to bind to IGF2BP2, we sought to determine whether circCD44 could affect Myc expression by regulating IGF2BP2. To this end, depletion of circCD44 decreased Myc expression, whereas ectopic expression of circCD44 enhanced Myc expression at both the RNA and protein levels (Fig. 7A, B, ***, *p* < 0.001). Given the roles of IGF2BP2 in stabilizing Myc mRNA and promoting its translation, we next performed a half-life assay and found that circCD44 knockdown indeed shortened the half-life of Myc mRNA (Fig. 7C, ***, *p* < 0.001). Furthermore, depletion of IGF2BP2 markedly decreased circCD44 ectopic expression-induced *Myc* mRNA levels, which could be abolished by reintroduction of WT IGF2BP2 but not the circCD44-binding deficient mutant (Fig. 7D, ***, *p* < 0.001). Similar results were obtained for Myc protein levels (Fig. 7E) and mRNA half-life (Fig. 7F, ***, *p* < 0.001) in the BT549 cell line. On the other hand, circCD44 Mut had no effect on the stability of C-Myc at either the RNA or protein level (Fig. 7D-F). To further confirm whether the function of the m6A reader of IGF2BP2 was engaged in the regulation of C-Myc, we first detected IGF2BP2 expression in cell lines with the indicated modifications. CircCD44 knockdown did not affect the expression of IGF2BP2 (Fig. 7G). We next transfected Flag-C-Myc (CRD domain included) into circCD44 knockdown cells to exclude the influence of endogenous C-Myc. The RIP assay was then applied using a m6A antibody, and m6A and C-Myc were detected (Fig. 7H, left). We found that stable circCD44 knockdown reduced the m6A modification of C-Myc mRNA compared with that in scramble control cells (Fig. 7I, left ***, *p* < 0.001). We next transfected IGF2BP2-specific shRNA into circCD44-overexpressing cells, and an RIP assay was applied (Fig. 7H, right). The results indicated that in circCD44-overexpressing cells, knocking down IGF2BP2 reduced m6A-modified C-Myc mRNA levels (Fig. 7I, right ***, *p* < 0.001). Taken together, our findings suggest that circCD44 can directly bind to IGF2BP2, a m6A reader, to enhance C-Myc expression.

The dysregulation of circRNAs in cancer has many causes. Given the critical role of circCD44 in TNBC, we also tried to determine upstream regulators of circCD44. The Alu sequence upstream and downstream in gene DNA promotes the formation of circRNAs [14]. Knupp et al. reported that RNA binding protein (RBP) could promote the formation of circRNAs [20]. We hypothesized that RBP could bind to CD44 premRNA, thus altering the splicing pattern and enhancing circCD44 formation. We noticed that ESRP1 and PCBP1 were reported as potential RBPs that promote circCD44 formation [21, 22]. We then detected the expression of ESRP1 and PCBP1 in the TCGA database and our in-house database, respectively. ESRP1 was upregulated in both the TCGA database and our in-house database (Supplementary Fig. 4A, B, *, *p* < 0.05, ***, *p* < 0.001), while PCBP1 showed no significant difference between tumor and normal tissues. Regression analysis between ESRP1 and circCD44 showed that ESRP1 was positively correlated with circCD44 (Supplementary Figure 4C, *r* = 0.81, ***, *p* < 0.001). We next established ESRP1 knockdown cell lines and overexpressed cell lines and subjected them to qRT–PCR to detect circCD44 using junction-specific primers. The results showed that circCD44 decreased in ESRP1 knockdown cell lines but increased in ESRP1-overexpressing cells (Supplementary Figure 4D, ***, *p* < 0.001). The above data suggested that ESRP1 promoted circCD44 expression in TNBC.

### CircCD44 promotes the tumorigenesis and progression of TNBC in vivo

**Fig. 8:**
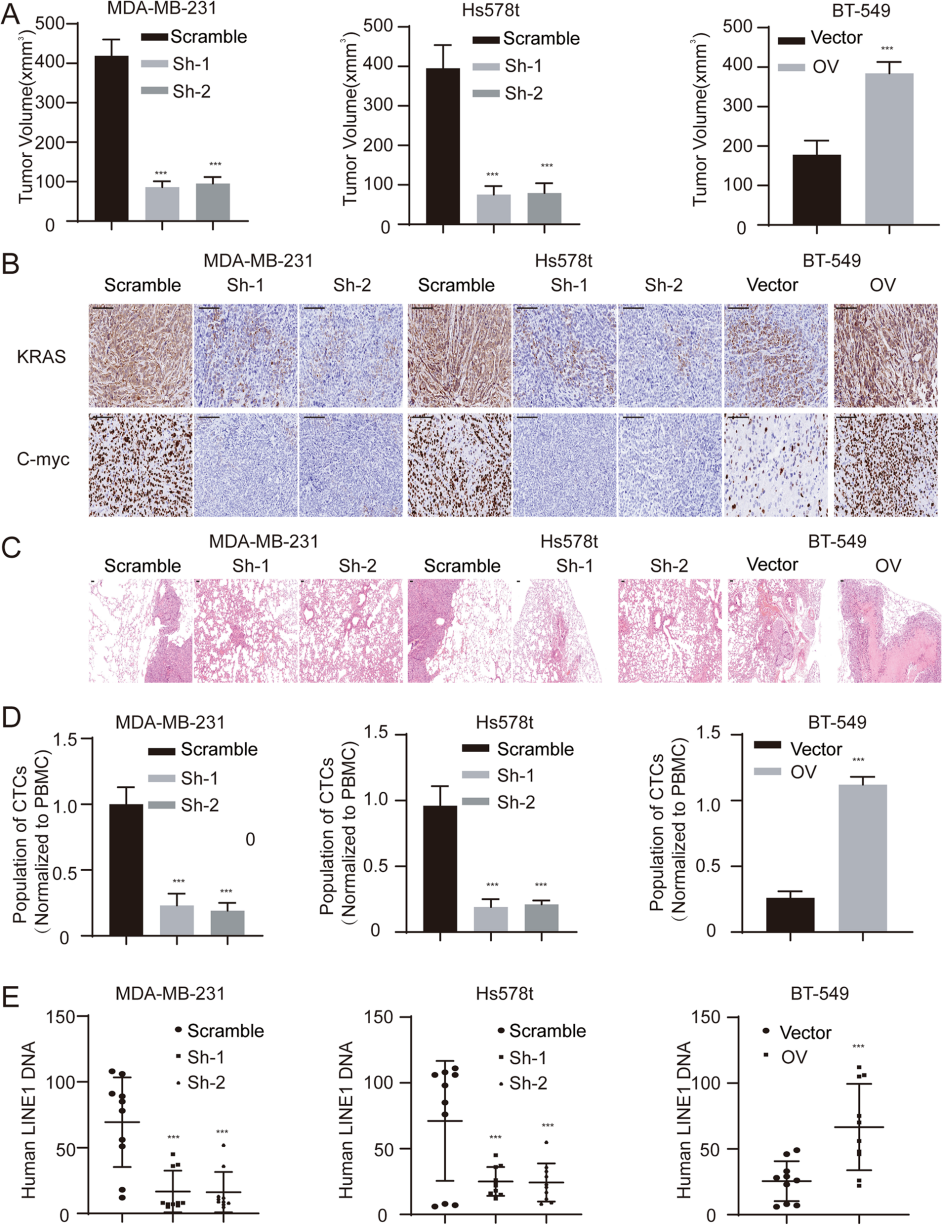
CircCD44 promotes tumorigenesis and progression of TNBCs in vivo. **A** Tumor volume of subcutaneous xenografts with the indicated modifications; ***, *P* < 0.001. **B** Subcutaneous xenograft tumors were collected, and KRAS and C-Myc were measured using IHC; scale bar: 200 μm. **C** Pulmonary tissue was harvested and subjected to HE staining, and a representative image of pulmonary metastatic tumors is shown; scale bar: 200 μm. **D** Blood was collected from mice, and the cycling tumor cells were identified using flow cytometry. The level of CTCs was normalized to that of peripheral blood monocytes; ***, *P* < 0.001. **E** Blood was collected, and human LINE1 DNA was detected and normalized to mouse MEFs; ***, *P* < 0.001. Data are representative of at least 2–3 experiments with similar results

To further study the in vivo functions of circCD44, we employed both xenograft models and lung metastasis models. The results showed that the depletion of circCD44 inhibited tumor growth, whereas ectopic expression of circCD44 facilitated tumor growth (Fig. 8A, Supplementary Figure 5A). The level of miR-502–5p increased in circCD44 knockdown tumors but decreased in circCD44-overexpressing tumors (Supplementary Figure 5B, ***, *p* < 0.001). More importantly, the expression of KRAS and Myc was positively correlated with the circCD44 level in these tumor tissues (Fig. 8B). Furthermore, we also observed that circCD44 expression was positively correlated with breast cancer lung metastasis (Supplementary Figure 5C), as detected through the H&E staining of lung cells (Fig. 8C), flow cytometry analysis of circulating tumor cells and RT–qPCR detection of human LINE1 (Figs. 8C-E, ***, *p* < 0.001). These findings indicate that circCD44 functions as an oncogenic circRNA to promote TNBC growth and metastasis.

## Discussion

CircRNAs are the most recently identified key players in TNBCs. circRNAs can act as competing endogenous RNAs to sponge miRNAs, to act as protein scaffolds, or to act as translational templates. These multiple functions suggested the critical roles of circRNAs during tumorigenesis and progression in TNBCs. Combination therapy was the most reliable for cancer therapy in TNBCs [23, 24]. Generally, different reagents target different molecules in the same pathway to avoid drug resistance. However, TNBCs still develop acquired drug resistance when targeting one pathway, and the gene status involved in other pathways affects the efficiency of targeted therapy [25]. In our study, we identified an uncharacterized circRNA - circCD44 - that was upregulated in TNBCs and promoted the progression of TNBCs by targeting both the miR-502-5p-KRAS axis and C-myc. Due to its dual targeting effects on KRAS and C-myc, targeting circCD44 in combination with other therapeutic approaches may not only achieve better therapeutic efficiency but also minimize resistance.

KRAS was reported to participate in the tumorigenesis and progression of breast cancers [26], and it plays a central role in the activation of many pathways, such as AKT/MEK/ERK signaling. Researchers have shown that overactivation of the KRAS pathway occurs in TNBCs and causes chemoresistance [27]. In recent decades, many efforts have been made to improve KRAS inhibitors. However, to date, only two compounds are currently in phase I clinical trials, highlighting the difficulties of this approach. Apart from KRAS inhibitors, in recent years, studies targeting the KRAS gene instead of the KRAS protein have attracted increasing attention. The G-quadruplex DNA sequence in the promoter region was reported to be essential for the transcription of KRAS. This region can be recognized by many transcription factors, such as MYC, which promote its downstream transcription [28]. miRNAs targeting the 5′ untranslated region of KRAS were reported to reduce the level of KRAS and thus inhibit the progression of TNBCs [29]. However, none of these miRNAs were reported to meet the clinical requirements, and more targets are still urgently needed. Our research indicated that circCD44 promotes KRAS expression by inhibiting miR-502–5p-induced KRAS degradation and that targeting circCD44 could be an attractive future approach to manipulate aberrant KRAS signaling in TNBCs.

C-Myc participates in physiological progression, including proliferation, differentiation and apoptosis [30]. C-Myc also plays central roles in the tumorigenesis and progression of TNBCs [31]. This centrality is due to its vital role in normal cells and cancer cells. Targeting C-Myc is a challenge but is still an urgent requirement [32]. The C-Myc-targeting strategy can be summarized as follows: 1) Targeting C-Myc transcription: stabilization of G-quadruplexes with small molecules such as QN-1 [33]. 2) Targeting C-Myc translation, such as targeting mTORC1 [34] and eIF4A [35] or destabilizing mRNA stability [36]. 3) Targeting C-Myc stability [37, 38]. 4) Targeting C-Myc downstream genes [38]. To date, no molecules have been applied in clinical practice. Our research provides a new choice for targeting C-Myc by affecting mRNA stability by modulating IGF2BP2 activity. As an m6A reader, IGF2BP2 was reported to determine the fate of mRNAs and to be associated with the methylation of mRNA, thus influencing mRNA stability. IGF2BP2 was also reported to be involved in the progression of numerous cancers, and it was recently suggested as a potential biomarker predicting prognosis [39]. The overexpression of IGF2BP2 may lead to the aberrant expression of many genes, including C-Myc [15, 40]. IGF2BP2 was also reported to regulate the expression of glycolysis genes and thus switch cancer metabolism to adapt to environmental changes [40]. In TNBCs, IMP2 and IMP3 were reported to cooperate to stabilize IGF2BP2 and thus contribute to metastasis [41–43]. However, although the function of IGF2BP2 is vital and indispensable, the crosstalk of IGF2BP2 and circRNAs in TNBCs is still unknown. We showed that IGF2BP2 bound to circCD44 was functional and stabilized C-Myc mRNA, which further explained the complex role of IGF2BP2 in TNBCs and re-enforced the critical role of circCD44 in TNBCs.

## Conclusion

We identified a new circRNA - circCD44 - through high-throughput sequencing. CircCD44 was upregulated only in TNBCs but not in other types of breast cancers. Its expression was negatively correlated with prognosis, indicating that it may serve as a novel and specific independent biomarker for TNBCs. CD44 is a stem marker of breast cancer and is very difficult to target [44]. In contrast to CD44, circCD44 promotes the progression of TNBC via both the miR-502–5p-KRAS and IGF2BP2-Myc axes. Targeting circCD44 decreased the expression of KRAS and C-myc, both of which are key drivers of TNBCs, highlighting the potential therapeutic role of circCD44 in TNBCs.

## Supplementary Information

**Additional file 1.**

## Data Availability

Raw sequencing and processed RNA Seq data from this study have been deposited into NCBI: SRP266211.

## Abbreviations

TNBC: Triple Negative Breast Cancer
circRNA: Circular RNA
ER: Estrogen receptor
PR: Progesterone receptor
HER2: Human epidermal growth factor receptor 2
PI3K: Phosphoinositide 3-Kinase
CDKs: Cyclin-dependent Kinases
RTKs: Receptor Tyrosine Kinases
FDA: Food and Drug Administration
MiRNAs: MicroRNAs
LncRNAs: Long noncoding RNA
circCD44: Circular CD44
ORF: Open reading frame
IRES: Internal ribosomal entry site
RBP: RNA-Binding Protein
TCGA: The Cancer Genome Atlas
FISH: RNA fluorescence in situ hybridization
