# Supplementary material for: CircCD44 plays oncogenic roles in triple-negative breast cancer by modulating the miR-502–5p/KRAS and IGF2BP2/Myc axes
Source: Mol Cancer. 2021 Oct 25;20:138. doi: 10.1186/s12943-021-01444-1 (PMC8543802; doi:10.1186/s12943-021-01444-1)
Supplement: Supplementary file 1 — Additional file 1. [file 12943_2021_1444_MOESM1_ESM.zip › Supplementary Figure Legends .docx]

**CircCD44 plays oncogenic roles in triple-negative breast cancer by modulating the miR-502-5p/KRAS and IGF2BP2/Myc axes**

Jie Li, Xinya Gao, Zhangqiang Zhang, Yuanhui Lai, Xunxun Lin, Bo Lin, Maoguang Ma, Xiaoli Liang, Xixi Li, Weiming Lv, Ying Lin and Nu Zhang

**SUPPLEMENTARY FIGURES AND LEGENDS**


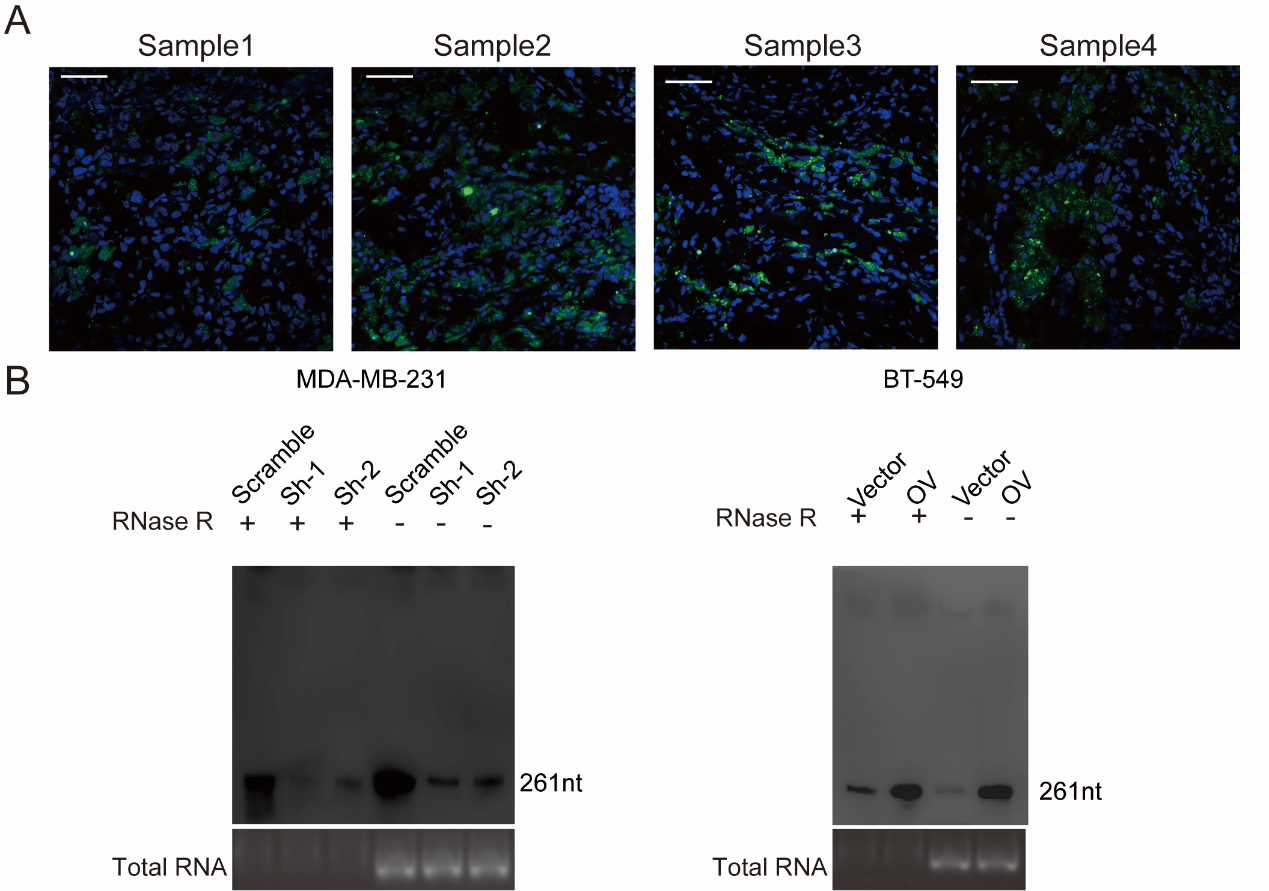


**Supplementary Figure 1. Identification of circCD44.**

**A.** 4 TNBCs were randomly picked and subjected to FISH using the junction specific probe. The representative images were shown, scale bar: 200 μm.

**B.** Total RNA of cells with indicated modifications were collect and RNase R was applied. Northern blot was applied to detect circCD44.

Data are representative of at least 2–3 experiments with similar results.


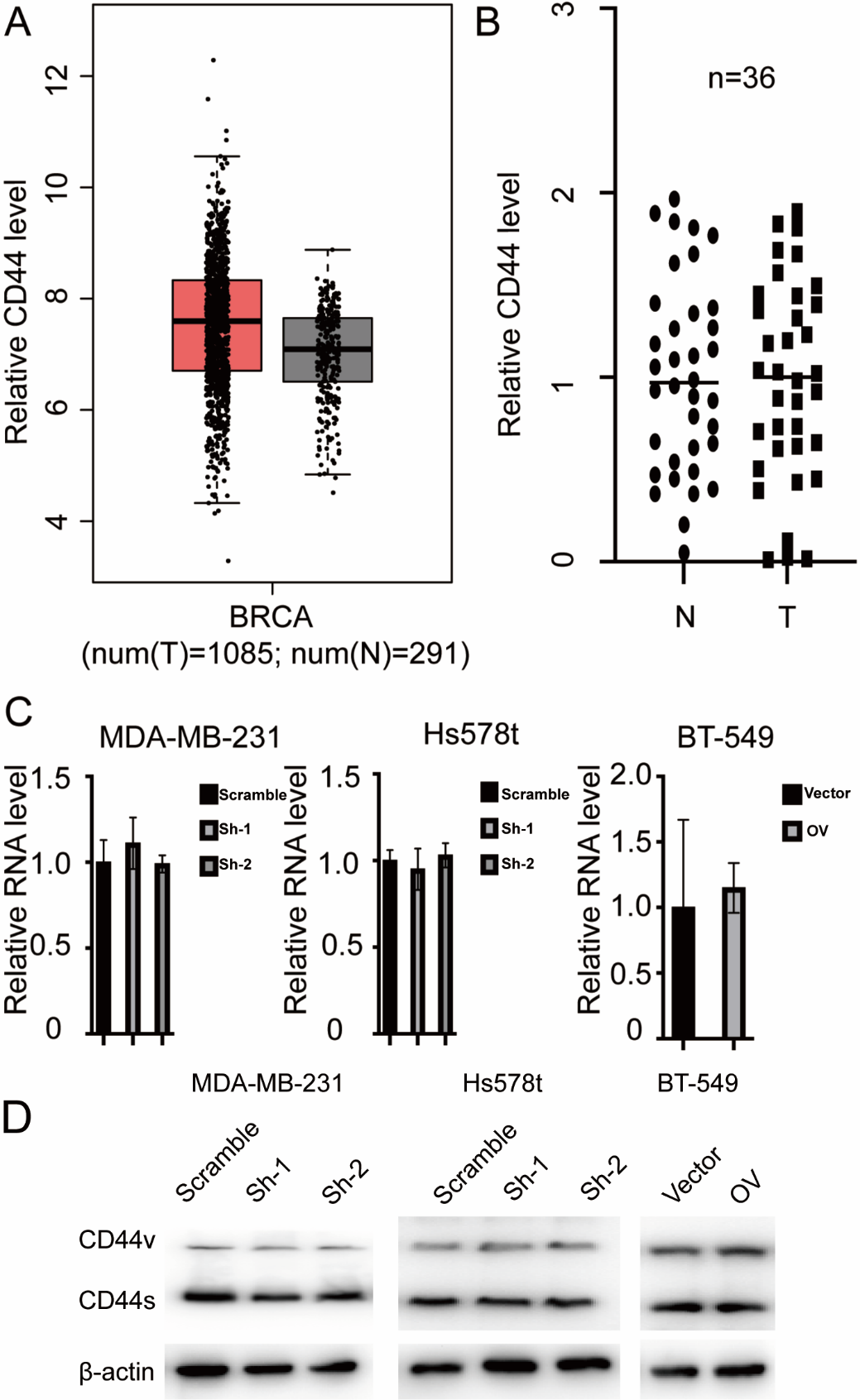


**Supplementary Figure 2. CircCD44 and linear CD44 perform their functions in different ways.**

**A.** The expression pattern of CD44 in the TCGA database.

**B.** The relative level of CD44 in our in-house database, 36 TNBCs were enrolled and the level of CD44 was detected and normalized.

**C.** The relative level of CD44 in cells with the indicated modifications.

**D.** The immunoblot of CD44s (CD44 standard) and CD44v (CD44 variant) in cells with the indicated modifications.

Data are representative of at least 2–3 experiments with similar results.


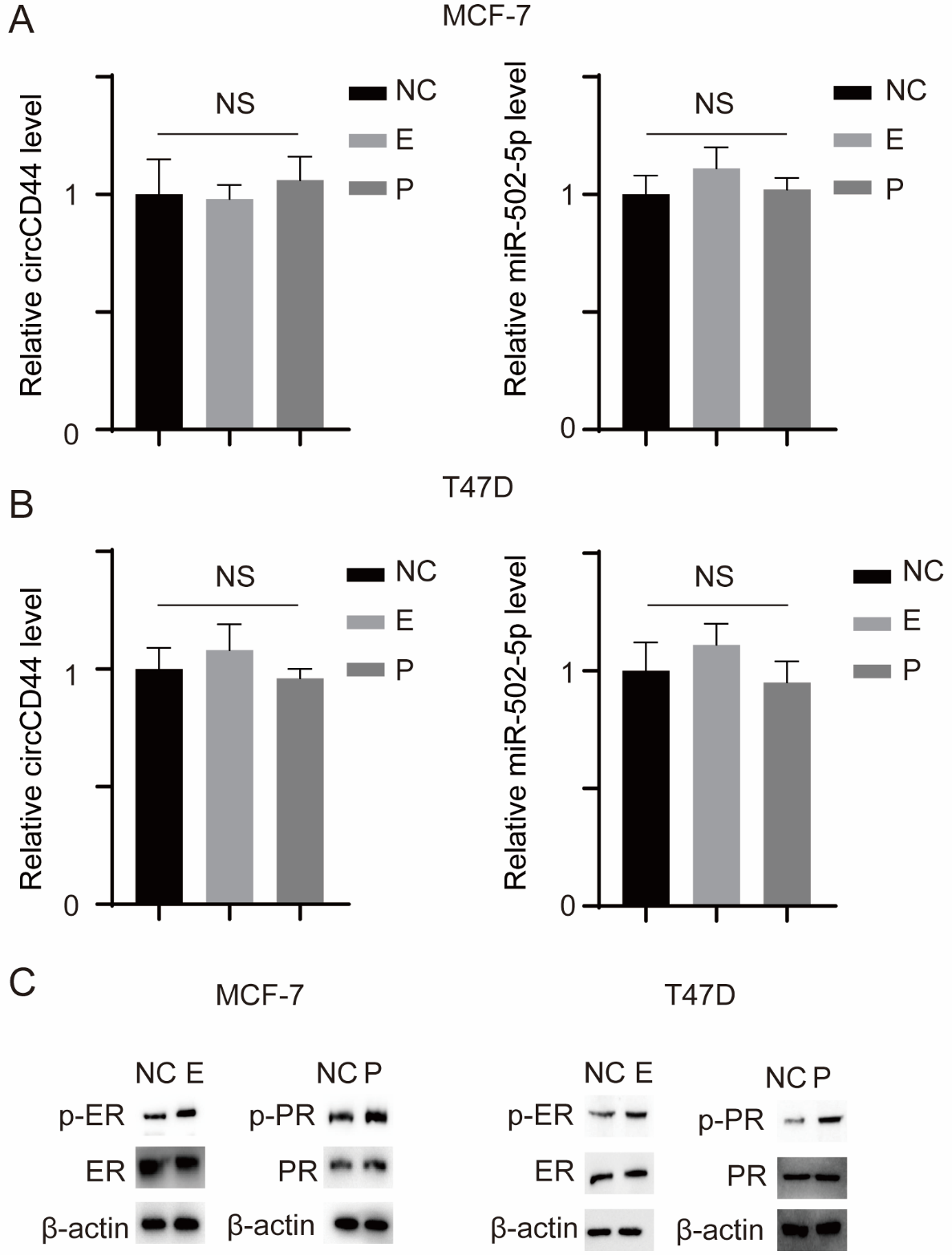


**Supplementary Figure 3. hormonal signaling didn’t influence the express of circCD44 and miR-502-5p.**

MCF-7 (A) and T47D (B) was treated with Estrone and Norgestimate (10mol/L each) and the expression of circCD44 and miR-502-5p was detected and normalized, NS, not significance, p-ER and p-PR were detected for conformation(C).

Data are representative of at least 2–3 experiments with similar results.


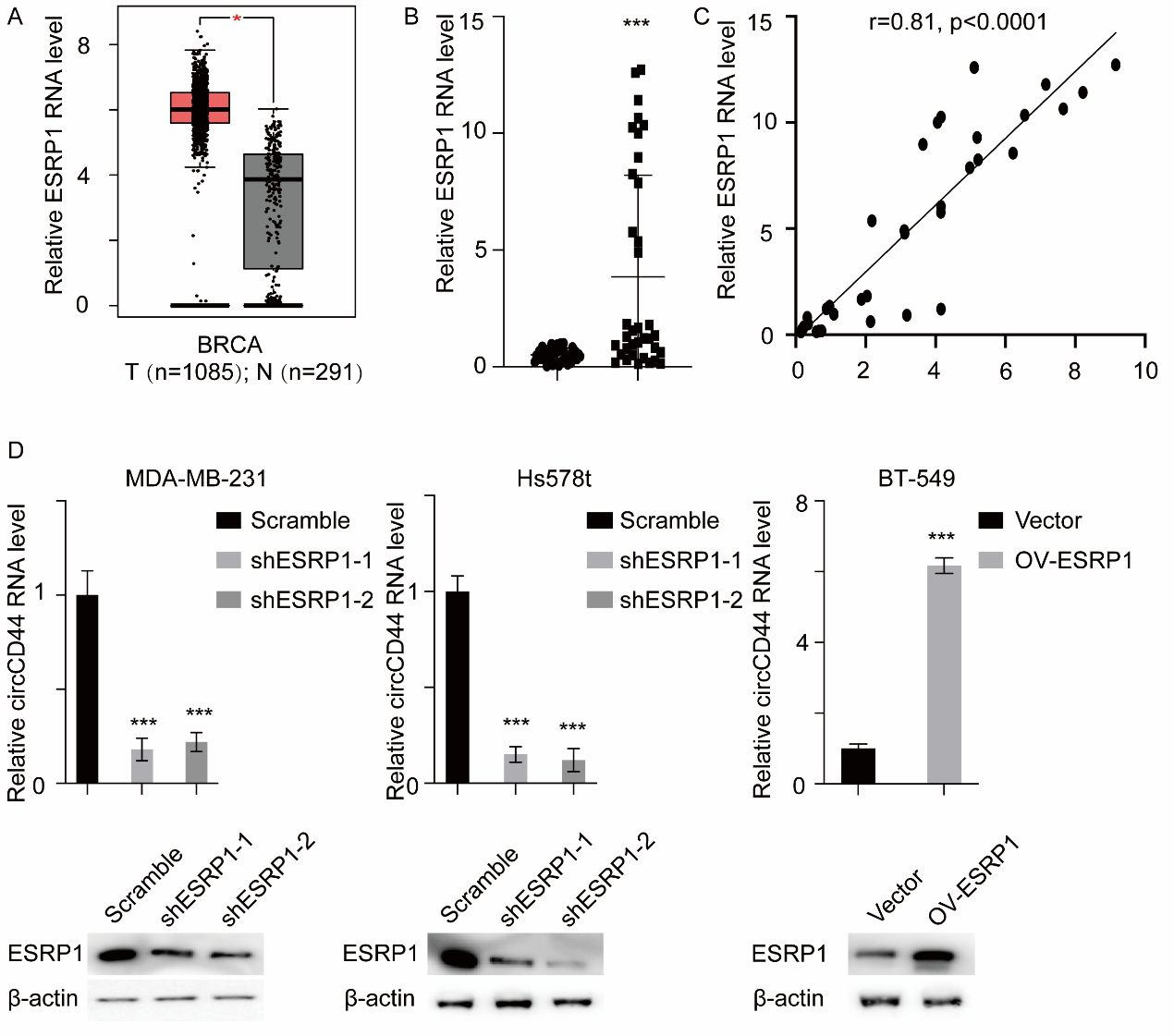


**Supplementary Figure 4. ESRP1 promotes the formation of circCD44.**

**A.** The expression pattern of ESRP1 in breast cancers in the TCGA database, *, P<0.05.

**B.** The relative level of ESRP1 in our in-house database, 36 TNBCs were subjected to qRT-PCR analysis, ESRP1 was detected and normalized, ***, P<0.001.

**C.** The regression analysis of ESRP1 and circCD44 of our in-house 36 TNBC patients.

**D.** ESRP1 stable knocking down cell lines and overexpress cell lines were established. CircCD44 was detected by qRT-PCR assay (upper, ***, P<0.001) and ESRP1 was detected using immunoblot for confirmation (lower).

Data are representative of at least 2–3 experiments with similar results.


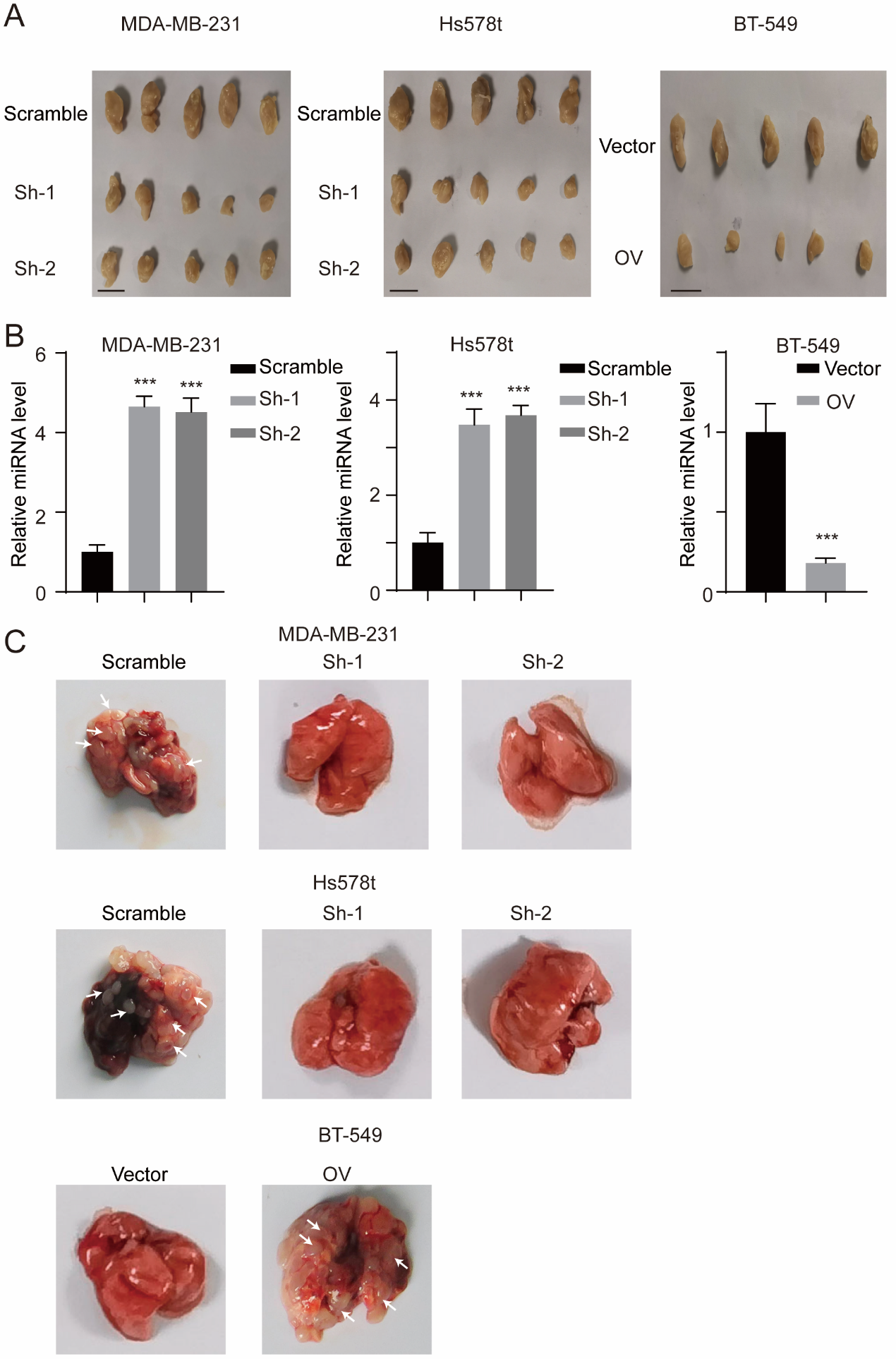


**Supplementary Figure 5. CircCD44 promoted the progression of TNBC *in vivo*.**

**A.** The subcutaneous xenograft implanted with indicated TNBCs. Scale bar, 1cm.

**B.** The relative level of miR-502-5p in the tumors collected from A, ***, P<0.001.

**C.** Representative images of lung metastatic assay, the arrows indicated the metastasis nodule.

Data are representative of at least 2–3 experiments with similar results.
